# Primary Sarcomas of the Larynx: A Case Series of Four Different Histopathologic Types

**DOI:** 10.1055/s-0043-1775997

**Published:** 2023-12-11

**Authors:** Ala S. Abu-Dayeh, Khaled A. Murshed, Adham Ammar, Mahir Petkar

**Affiliations:** 1 Division of Anatomic Pathology, Hamad Medical Corporation, Doha 576214, Qatar

**Keywords:** larynx, sarcoma, chondrosarcoma, leiomyosarcoma, liposarcoma, undifferentiated pleomorphic sarcoma

## Abstract

Primary laryngeal sarcomas are rare. Their nomenclature and classification are similar to soft tissue counterparts; however, there are notable differences between clinical presentation, behavior, treatment, and follow-up. There is sparse information regarding the clinical features, biologic behavior, and treatment modalities of laryngeal sarcomas. To increase our understanding about these tumors, we describe herein an additional series of four cases of different pathologic types of laryngeal sarcomas, including low-grade chondrosarcoma, leiomyosarcoma, well-differentiated liposarcoma, and undifferentiated pleomorphic sarcoma. Our main aim is to upsurge awareness about the morphologic variations of laryngeal sarcomas, to avoid potential pitfalls during histopathologic examination. It is essential to ensure that correct diagnosis, subclassification, and grading are achieved for proper guidance of treatment and clinical follow-up at multidisciplinary team meetings.

## Introduction

Laryngeal neoplasms are divided into epithelial and nonepithelial tumors based on their origin. 1 2 Epithelial tumors comprise the majority of laryngeal neoplasms, and squamous cell carcinoma is the most common malignant neoplasm of the larynx. 1 2 3 Nonepithelial tumors are much less common. This category includes sarcoma, melanoma, and lymphoma. Primary laryngeal sarcomas are rare and account for less than 1% of laryngeal tumors. 1 2 3 4 5 They are classified based on their phenotype and histological grade, similar to their soft tissue counterparts. However, the clinical presentation, treatment, and prognosis of laryngeal sarcomas are different. 1 2 3 4 There are few studies of laryngeal sarcomas reported in the literature. 1 2 3 4 5 Despite that, information regarding the clinical features, biologic behavior, and treatment modalities of laryngeal sarcomas is still limited. Herein, we describe an additional four cases of different pathologic types of laryngeal sarcomas, to increase awareness and understanding about these rare tumors, to avoid potential diagnostic pitfalls.

## Case Presentation

### Case 1

**Fig. 1:**
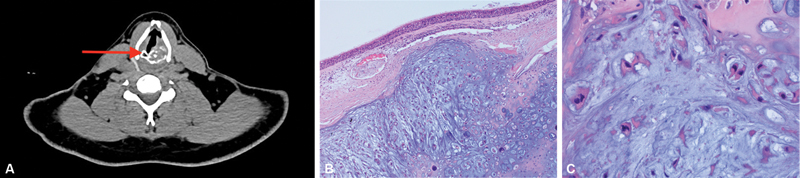
Radiological and microscopic features of low-grade chondrosarcoma. ( **A** ) Computed tomography scan of the neck demonstrates a lesion in the infraglottic (red arrow). ( **B** ) Photomicrograph shows lobule of neoplastic hyaline cartilage (hematoxylin and eosin [H&E] stain, 100x). ( **C** ) High-power view demonstrates increased cellularity, and mild nuclear atypia, (H&E stain, 400x).

A 49-year-old male presented to the emergency department with difficulty in breathing and weight loss for 3-month duration, exacerbated by physical activity. Fiberoptic examination revealed a subglottic fleshy soft tissue mass obstructing 80% of the upper airway. Computed tomography (CT) scan of the neck revealed a soft tissue mass lesion in the infraglottic region at the left side of cricoid and arytenoids cartilage, protruding inside the lumen and compromising the airway ( Fig. 1A ). The patient underwent total laryngectomy with left hemithyroidectomy. Macroscopic examination of the resected specimen revealed a submucosal ill-defined tan lobulated mass, occupying the left thyroid laryngeal cartilage that measured 2.9 × 2.5 × 2 cm. Microscopic examination showed a tumor composed of lobules of hyaline cartilage permeating into the adjacent bony trabeculae. The tumor showed mild increase in cellularity as well as cytological atypia, in the form of nuclear hyperchromasia and occasional binucleation ( Fig. 1B and C ). The diagnosis of low-grade chondrosarcoma was rendered. The resection margin was free of the tumor. Postoperative course was uneventful. Five months later, there was no evidence of tumor recurrence or metastasis by imaging studies.

### Case 2

**Fig. 2:**
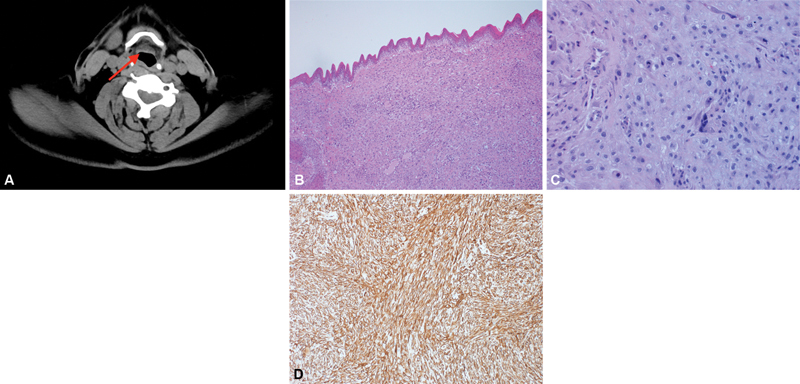
Radiological, microscopic, and immunohistochemical features of leiomyosarcoma. ( **A** ) Computed tomography scan of the neck demonstrates thickening at the glottic anterior commissure, with focal submucosal nodular lesion (red arrow). ( **B** ) Photomicrograph shows submucosal fascicles of moderately atypical spindle cells (hematoxylin and eosin [H&E] stain, 100x). ( **C** ) High-power view shows moderately atypical spindle cells proliferation (H&E stain, 400x). ( **D** ) The tumor cells demonstrate diffuse, strong cytoplasmic, and membranous reactivity for smooth muscle actin.

A 57-year-old male patient presented with hoarseness of voice for one-year duration. A glottic mass arising from the right vocal cord was found by fiberoptic examination. CT scan of the neck showed thickening at the glottic anterior commissure, with focal submucosal nodular intense enhancing lesion ( Fig. 2A ). The patient underwent right anterior cordectomy and debulking of the glottic mass. Macroscopic examination of the resected specimen revealed multiple tan/white irregular rubbery and friable soft tissue fragments, measuring 3 × 1.5 × 0.5 cm. Histopathological examination showed a submucosal tumor composed of fascicles of moderately atypical spindle cells with abundant eosinophilic cytoplasm, marked nuclear pleomorphism, and prominent hyperchromasia. Brisk mitotic activity was noted ( Fig. 2B and C ). The tumor cells were strongly and diffusely reactive for smooth muscle actin (SMA) ( Fig. 2D ) and calponin, but negative for keratin AE1/AE3, keratin MNF 116, keratin ⅚, desmin, MyoD1, myogenin, S100, and Melan A. Based on these findings, the diagnosis of leiomyosarcoma was made. The resection margin was free of the tumor. Two weeks after the surgery, the patient noticed improvement of his voice. Ten months later, there was no evidence of recurrence or metastasis by imaging studies.

### Case 3

**Fig. 3:**
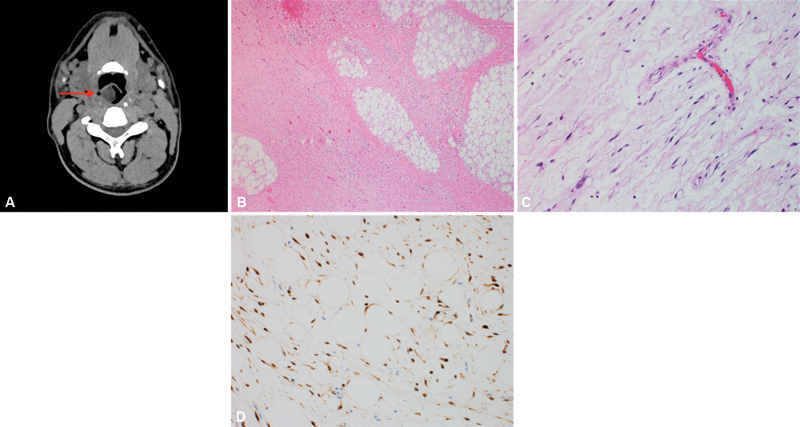
Radiological, microscopic, and immunohistochemical features of well-differentiated liposarcoma. ( **A** ) Computed tomography scan of the neck shows a well-defined lesion along the posterior wall of the hypopharynx (red arrow). ( **B** ) Photomicrograph shows lobules of adipose tissue separated by thick fibrous septa containing atypical hyperchromatic spindle cells (hematoxylin and eosin [H&E] stain, 40x). ( **C** ) High-power view shows atypical hyperchromatic spindle cells in a prominent myxoid stroma with arborizing chicken-wire vasculature (H&E stain, 200x). ( **D** ) The tumor cells demonstrate nuclear staining for CDK4.

A 36-year-old gentleman presented to the outpatient clinic with difficulty of breathing and swallowing for 3-month duration. Fiberoptic examination revealed a smooth mobile swelling arising from the right hypopharyngeal wall causing partial airway obstruction. CT scan of the neck revealed a lesion along the posterior wall of the hypopharynx ( Fig. 3A ). Macroscopic examination of the resected specimen revealed a nodular rubbery lesion measuring 3.5 × 3 × 1.5 cm. Microscopic examination showed a submucosal tumor composed of lobules of atypical spindle cells in a prominent myxoid stroma with arborizing chicken-wire vasculature. Other areas showed lobules of adipose tissue separated by thick fibrous septa that contains atypical hyperchromatic spindle cells ( Fig. 3B and C ). By immunohistochemistry, the tumor cells were reactive for CDK4 ( Fig. 3D ). By fluorescence-in-situ-hybridization (FISH) analysis, *MDM2* gene amplification was detected along with amplification of DDIT3 probe region. Based on the immunohistochemistry and cytogenetics results, the diagnosis of well-differentiated liposarcoma (WDL) with prominent myxoid stroma was rendered. There was no evidence of tumor recurrence or metastasis detected by magnetic resonance imaging performed 6 months later.

### Case 4

**Fig. 4:**
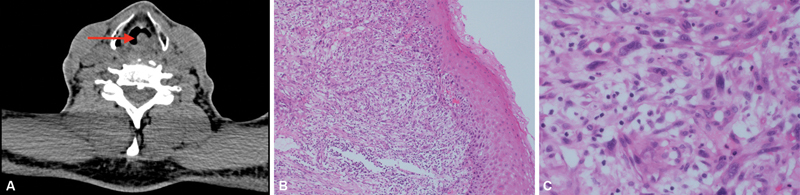
Radiological and microscopic features of undifferentiated pleomorphic sarcoma. ( **A** ) Computed tomography scan of the neck demonstrates a heterogeneous enhancing laryngeal lesion (red arrow). ( **B** ) Photomicrograph demonstrates submucosal malignant spindle cell neoplasm (hematoxylin and eosin [H&E] stain, 100x). ( **C** ) High-power view shows pleomorphic and bizarre spindle tumor cells (H&E stain, 400x).

A 59-year-old ex-smoker, male patient presented with hoarseness of voice, stridor, and shortness of breath for 3 months duration. Fiberoptic examination revealed no obvious masses. CT scan of the neck showed a heterogeneous enhancing laryngeal mass, partially obstructing the upper airway ( Fig. 4A ). The patient underwent total laryngectomy with bilateral neck dissection. Macroscopic examination of the resected specimen showed a tan-white irregular mass, located at the glottis, involving the left true vocal cord, and extending to the midline that measures 1.5 × 0.8 × 0.7 cm. Microscopic examination showed extensive infiltration by a malignant neoplasm, composed of pleomorphic and bizarre spindle cells, interspersed by numerous histiocytes ( Fig. 4B and C ). Marked mitotic activity was identified, including atypical forms. A wide panel of immunohistochemical stains was performed, all of which were negative except vimentin. CD68 was reactive in the histiocytic population. Based on these findings, the diagnosis of undifferentiated pleomorphic sarcoma was rendered. The tumor was completely excised. One month later, radiotherapy was initiated. He was followed up at 6- and 12-month intervals and there were no signs of recurrence or metastasis.

## Discussion

Laryngeal neoplasms are divided into epithelial and nonepithelial tumors. 1 2 The vast majority of laryngeal tumor are carcinomas, and squamous cell carcinoma is the most common histologic type. 1 2 3 4 5 Nonepithelial tumors constitute approximately 5% of all laryngeal neoplasms that include sarcoma, lymphoma, and melanoma. 1 2

Laryngeal sarcomas account for less 1% of laryngeal neoplasms. 1 2 3 4 5 Various sarcoma types have been reported to occur in the larynx. Their nomenclature and classification are similar to their soft tissue counterpart. However, there are considerable differences between laryngeal sarcomas and soft tissue sarcomas. The former presents early and are diagnosed at earlier stage. Thus, laryngeal sarcomas have lower mortality and less rate of local recurrence and distant dissemination. 1 2 In addition to that, negative margins are difficult to achieve in laryngeal sarcomas due to the anatomical complexity of the region. Moreover, laryngeal tumors are removed in piecemeal in some cases, which impose diagnostic challenge for the pathologist examining the specimen.

There is wide variation in the histomorphologic features of laryngeal sarcomas. Chondrosarcoma is the most common primary sarcoma of the larynx. 1 6 7 8 It has been found that the conventional subtype is the most common; however, other subtypes such as clear cell, mesenchymal and dedifferentiated chondrosarcomas have been reported. 6 7 8 More than 50% of cases arise in association with chondroma component, which makes the diagnosis of low-grade chondrosarcoma more challenging. 1 6 7 Tumor cellularity and nuclear atypia are useful features to differentiate chondroma from low-grade chondrosarcoma, the latter is more cellular, and the chondrocytes demonstrate nuclear atypia in the form of hyperchromasia and irregular nuclear contours with frequent binucleation. Extensive sampling of the tumor is necessary to detect any dedifferentiated component, which is characterized by the presence of high-grade spindle cell sarcoma adjacent to the low-grade component. 1 6 7 Up to 50% of laryngeal chondrosarcomas develop local recurrence, and the risk increases with incomplete excision. 6 7 However, they have very low risk of distant metastasis. 6 7 Therefore, conservative surgery with preservation of the laryngeal function and obtaining negative margins is advocated. In the largest cohort study of laryngeal chondrosarcoma, they found that it has an excellent prognosis with relatively high 5- and 10-year survival rates (88 and 66%, respectively). 8

Leiomyosarcoma of the larynx is much less common. 1 9 It most commonly arises from the glottic and supraglottic regions. 1 9 It is essential to rule out the possibility of sarcomatoid carcinoma before making the diagnosis of leiomyosarcoma. Immunohistochemical stains are useful in this setting; sarcomatoid carcinomas are reactive for high molecular weight keratin, p63 and p40. On the other hand, leiomyosarcoma would be negative for these antibodies, but positive for smooth muscle markers such as smooth muscle myosin, desmin, and caldesmon. 10 Another important feature that helps to distinguish sarcomatoid carcinoma is the presence of dysplasia in the overlying surface squamous epithelium. Patients with laryngeal leiomyosarcoma have been found to be at increased risk for distant dissemination 11 therefore, surgical resection with negative margins, in addition to adjuvant chemotherapy, should be considered. 9 10

Liposarcomas of the larynx are usually well-differentiated, either in the form of lipoma-like or sclerosing subtypes. 12 It is essential to differentiate WDL from lipoma and its variants, such as spindle cell and pleomorphic lipoma. The presence of lipocytes of variable sizes, thick fibrous bands that traverse the tumor, and atypical hyperchromatic spindle cells are features that favor WDL. In some cases, with limited tissue material, the diagnosis is difficult to achieve on hematoxylin and eosin stain. In such cases, the use of MDM2 and CDK4 by immunohistochemistry and/or FISH is helpful to establish the diagnosis. 13 14 Positive staining for MDM2 and/or CDK4 or the detection of their amplified genes by FISH confirms the diagnosis of WDL. 13 14 It is also essential to extensively sample the tumor to rule out the possibility of dedifferentiated liposarcoma, which is characterized by the presence of high-grade spindle cell sarcoma adjacent to the well-differentiated component. 12 It has been found that up to 50% of laryngeal liposarcomas recur; therefore, the current recommendation is to perform wide surgical excision with negative margins. 1 12

Undifferentiated pleomorphic sarcoma (UPS) is a rare laryngeal tumor that arises most commonly from the glottis. 15 It is a diagnosis of exclusion that is made after running a wide panel of immunohistochemical stains and ancillary studies. Sarcomatoid carcinoma is essential to exclude, which is usually positive for high molecular weight keratin, p63 and p40. Dedifferentiated liposarcoma is another possibility that should be considered and ruled out by running MDM2 and CDK4 through immunohistochemistry or FISH. UPS/MFH is an aggressive tumor with high rate of local recurrence and metastasis 16 17 18 therefore, wide surgical excision and obtaining negative margins are recommended. 17 Adjuvant radiotherapy and/or chemotherapy may be considered in large tumors and patients with positive margins. 16

In summary, we describe an additional series of four cases of primary laryngeal sarcoma ( Table 1 ), which adds on more information about this rare category of laryngeal tumors. The perplexity of resection of laryngeal tumors and difficulty to achieve negative margins may impose a diagnostic challenge. Correct identification of the histomorphologic features, and awareness of the various subtypes of laryngeal sarcomas, is essential to guide the treatment plan that is tailored for patients in tumor boards.

**Table 1:** Summary of the clinicopathological features of the primary laryngeal sarcomas

	Low-grade chondrosarcoma	Leiomyosarcoma	Well-differentiated liposarcoma	UPS
Age (years)/gender	49/Male	57/Male	36/Male	59/Male
Presentation	Difficulty in breathing and weight loss	Voice hoarseness	Difficulty in breathing and swallowing	Voice hoarseness, stridor, and SOB
Tumor location	Subglottis	Glottic anterior commissure	Posterior wall of the hypopharynx	Glottis and left true vocal cord
Tumor size (cm)	2.9 × 2.5 × 2 cm	3 × 1.5 × 0.5 cm	3.5 × 3 × 1.5 cm	1.5 × 0.8 × 0.7 cm
Treatment modality	Total laryngectomy with left hemithyroidectomy	Right anterior cordectomy and debulking of the mass	Mass excision	Total laryngectomy with bilateral neck dissection and radiotherapy
Margin status	Negative	Negative	Positive	Negative
Recurrence	No recurrence	No recurrence	No recurrence	No recurrence
Follow-up (months)	5 months No recurrence or metastasis.	10 months No recurrence or metastasis.	6 months No evidence of local recurrence or definite metastatic neck lymph nodes noted on MRI neck	12 months No recurrence or metastasis

Abbreviations: MRI, magnetic resonance imaging; SOB, shortness of breath; UPS, undifferentiated pleomorphic sarcoma.

## Declaration

Two cases were published previously separately as case reports. The undifferentiated pleomorphic sarcoma case was published under the title “Glottic Malignant Fibrous Histiocytoma: A Case Report and Literature Review” (Aljariri AA, Alsaleh AR, Al-Enazi HA, Haider HA, Petkar M, Rahman W, Nashwan AJ. Glottic Malignant Fibrous Histiocytoma: A Case Report and Literature Review. Case Rep Oncol. 2021 Mar 31;14(1):641–646. doi: 10.1159/000514977. PMID: 33976647; PMCID: PMC8077659). The WDL case was published under the title “Well-Differentiated Liposarcoma of the Hypopharynx Exhibiting Myxoid Liposarcoma-like Morphology with MDM2 and DDIT3 Co-Amplification” (Murshed KA, Abo Samra H, Ammar A. Well-Differentiated Liposarcoma of the Hypopharynx Exhibiting Myxoid Liposarcoma-like Morphology with MDM2 and DDIT3 Co-Amplification [published online ahead of print, 2021 Jun 4]. *Head Neck Pathol* . 2021;10.1007/s12105-021-01341-5. doi:10.1007/s12105-021-01341-5).

## References

[1] Velez Torres J M Martinez Duarte E Diaz-Perez J A Primary sarcomas of the larynx: a clinicopathologic study of 27 cases Head Neck Pathol 2021 15 03 905 916 33686585 10.1007/s12105-021-01314-8 PMC8384992

[2] Ramdulari A V Izzuddeen Y Benson R Mallick S Venkatesulu B Giridhar P Laryngeal soft tissue sarcoma: systematic review and individual patient data analysis of 300 cases Head Neck 2021 43 05 1421 1427 33448036 10.1002/hed.26604

[3] Astl J Holy R Tuckova I Belsan T Pala M Rotnagl J Sarcomas of the larynx: one institution's experience and treatment protocol analyses Medicina (Kaunas) 2021 57 03 192 33668739 10.3390/medicina57030192 PMC7996352

[4] AbdullGaffar B Keloth T Laryngeal sarcomas: a case series of 5 cases Ann Diagn Pathol 2018 37 35 41 30241033 10.1016/j.anndiagpath.2018.09.007

[5] Bayır Ö Karagöz T Han Ü A report of 4 laryngeal sarcoma patients JSM Clin Case Rep. 2016 4 03 1105 1109

[6] Thompson L D Gannon F H Chondrosarcoma of the larynx: a clinicopathologic study of 111 cases with a review of the literature Am J Surg Pathol 2002 26 07 836 851 12131151 10.1097/00000478-200207000-00002

[7] Coca-Pelaz A Rodrigo J P Triantafyllou A Chondrosarcomas of the head and neck Eur Arch Otorhinolaryngol 2014 271 10 2601 2609 24213203 10.1007/s00405-013-2807-3

[8] Talati V M Urban M J Patel T R Laryngeal chondrosarcoma characteristics and survival analysis in the National Cancer Database Otolaryngol Head Neck Surg 2022 166 01 101 108 33848444 10.1177/01945998211004578

[9] Marioni G Bertino G Mariuzzi L Bergamin-Bracale A M Lombardo M Beltrami C A Laryngeal leiomyosarcoma J Laryngol Otol 2000 114 05 398 401 10912277 10.1258/0022215001905698

[10] Marioni G Staffieri C Marino F Staffieri A Leiomyosarcoma of the larynx: critical analysis of the diagnostic role played by immunohistochemistry Am J Otolaryngol 2005 26 03 201 206 15858778 10.1016/j.amjoto.2004.11.007

[11] Sauter A Bersch C Lambert K L Hörmann K Naim R Chondrosarcoma of the larynx and review of the literature Anticancer Res 2007 27(4C):2925 2929 17695472

[12] Wenig B M Weiss S W Gnepp D R Laryngeal and hypopharyngeal liposarcoma. A clinicopathologic study of 10 cases with a comparison to soft-tissue counterparts Am J Surg Pathol 1990 14 02 134 141 2301699 10.1097/00000478-199002000-00005

[13] Binh M B Sastre-Garau X Guillou L MDM2 and CDK4 immunostainings are useful adjuncts in diagnosing well-differentiated and dedifferentiated liposarcoma subtypes: a comparative analysis of 559 soft tissue neoplasms with genetic data Am J Surg Pathol 2005 29 10 1340 1347 16160477 10.1097/01.pas.0000170343.09562.39

[14] Murshed K A Abo Samra H Ammar A Well-differentiated liposarcoma of the hypopharynx exhibiting myxoid liposarcoma-like morphology with MDM2 and DDIT3 co-amplification[published online ahead of print, 2021 Jun 4]Head Neck Pathol 2022 16 01 288 293 34089125 10.1007/s12105-021-01341-5 PMC9018935

[15] Jeong C Y Kim C S Undifferentiated pleomorphic sarcoma of the vocal fold Ear Nose Throat J 2016 95 12 E12 E14 27929601

[16] Haberal I Samim E Astarci M Ozeri C Radiation-induced malignant fibrous histiocytoma of the neck in a patient with laryngeal carcinoma Am J Otolaryngol 2001 22 02 146 149 11283832 10.1053/ajot.2001.22579

[17] Cambruzzi E Cruz R Gava V Pegas K Undifferentiated high-grade pleomorphic sarcoma of the larynx treated with partial laringectomy Rev Bras Otorrinolaringol (Engl Ed) 2020 86(Suppl 1, Suppl 1):14 16 10.1016/j.bjorl.2016.11.005 PMC9422573 28108272

[18] Aljariri A A Alsaleh A R Al-Enazi H A Glottic malignant fibrous histiocytoma: a case report and literature review Case Rep Oncol 2021 14 01 641 646 33976647 10.1159/000514977 PMC8077659

